# Morning Appointment Time Rather Than Training Load Affects Sleep During a Training Camp in Young Elite Rugby Union Players

**DOI:** 10.1002/ejsc.70020

**Published:** 2025-10-19

**Authors:** Maxime Chauvineau, Bertrand Mathieu, Gaëtan Boissard, Julien Piscione, François Duforez, Gaël Guilhem, Mathieu Nedelec

**Affiliations:** ^1^ Laboratory Sport, Expertise and Performance (EA 7370) French Institute of Sport (INSEP) Paris France; ^2^ Paris Cité University Paris France; ^3^ French Rugby Federation Marcoussis France; ^4^ Hotel‐Dieu, Sleep and Vigilance Center APHP Paris France

**Keywords:** elite athletes, recovery, sleep architecture, team sports, training program

## Abstract

This investigation aimed to evaluate sleep of young rugby union players during a 10‐day training camp accounting for the potential influence of prior daily training load and the morning appointment time. Twenty‐six elite male under‐20 rugby union players were monitored each day during a 10‐day training camp including two exhibitions matches. Sleep‐wake patterns and sleep architecture were assessed using actigraphy and a reduced‐montage dry‐electroencephalographic headband device, respectively. Training load and perceived wellness were, respectively, evaluated using GPS trackers and 10‐score visual analogue scales. The prevalence of nights with sleep duration < 7 h, wake after sleep onset > 40 min and sleep efficiency < 85% was 30.3%, 77.8% and 43.4%, respectively. Every 100‐m increase in high‐speed running distance increased sleep duration (*β* = +4.9 min, *p* < 0.05) and reduced the number of sleep stage shifts (*β* = −1.1, *p* < 0.05). The shortest sleep duration (06:52 ± 00:34 h) occurred on the day of Match 1, when the morning appointment was the earliest, that is, 7:30. Sleep duration (−19.3 min, *p* = 0.01) and efficiency (−2.2%, *p* < 0.01) were impaired when the morning appointment was scheduled at 8:30 compared to 8:00. This study supports that the training camp is a vulnerable period for sleep, but a controlled, non‐excessive training load promotes sleep quantity and continuity of sleep architecture. The organisational aspects of the camp strongly influence the sleep‐wake patterns. Coaches should be aware of the putative impact of earlier and/or unusual morning appointment times on sleep, especially in proximity to a match.

## Highlights

- Young elite rugby union players experienced difficulties in achieving adequate sleep quantity and had prolonged awakenings during the training camp.
- Increased daily high‐velocity running demands (i.e., distance > 18 km.h^−1^) resulted in longer subsequent sleep duration and enhanced the continuity of sleep architecture.
- It is recommended to propose consistent morning appointment time during a training camp to promote regular sleep timing and adequate sleep quantity and quality.

## 1 Introduction

Rugby union is an intermittent team‐sport, characterised by periods of high‐intensity running activities and collision‐based actions interspersed with low‐intensity activities (Till et al. 2020). The high‐intensity and combative nature of rugby leads to biochemical, metabolic and neuromuscular alterations that can result in fatigue and impaired physical performance (Tavares et al. 2017; Naughton et al. 2018, 2021). To adequately prepare for such substantial physical demands, elite rugby union teams undergo training camps including strenuous training sessions. In this context, it is essential that players achieve an adequate quantity and quality of sleep to facilitate recovery, adapt to training, and maintain well‐being and performance (Halson and Juliff 2017; O'Donnell et al. 2018).

Despite the importance of sleep, training camps have been identified as a vulnerable period for sleep in team‐based contact sports, with evidences of impairments in sleep‐wake patterns, that is, sleep duration, wake after sleep onset (WASO) and/or sleep efficiency (Pitchford et al. 2017; Thornton et al. 2017). One of the putative reasons suggested for these sleep disturbances is the high training load that players can experience (Roberts et al. 2019). Nevertheless, the detrimental effect of increased training load on rugby players' sleep is not consistent across studies, and some studies have shown that the rugby‐specific physical demands can have variable effects on sleep (Thornton et al. 2017, 2018; Leduc et al. 2019). In rugby league players, an increase in daily acceleration/deceleration efforts was associated with subsequent improvements in sleep duration and sleep efficiency, although higher daily distance covered and distance covered at high‐velocity (> 4 m.s^−1^) were detrimental to sleep duration and/or sleep efficiency (Thornton et al. 2017, 2018). However, these studies used subjective and/or actigraphy measurements to assess sleep‐wake patterns, and did not evaluate the internal structure of sleep, that is, sleep architecture, which is critical in determining sleep quality. To the best of our knowledge, only one study has highlighted the varied effects of the combative and high‐intensity intermittent demands of rugby union during a match on subsequent sleep architecture, using a single‐night polysomnography (Aloulou et al. 2020). Increased high‐velocity (> 18 km.h^−1^) running distance was associated with a subsequent reduction in the proportion of light sleep, in favour to higher proportions of slow‐wave sleep (SWS) and, conversely, increased number of collisions resulted in higher light sleep proportions. However, the putative effect of rugby‐specific physical demands on sleep architecture throughout a training camp has not yet been investigated.

Another important consideration for sleep difficulties during a camp is the often‐hectic program (Nedelec et al. 2018). Camp situations are often accompanied by a change in sleeping environment and unaccustomed schedules, with several training sessions a day, sometimes exhibition matches, and commitments outside of training (e.g., meetings, working sessions, collective lunches, travels). These environmental and organisational variations may affect sleep behaviours, that is, bedtime and get‐up time (Pitchford et al. 2017; Thornton et al. 2017). In particular, the morning schedule has been demonstrated to strongly determine the previous sleep duration in various sports (Sargent, Halson and Roach 2014; Sargent, Lastella, et al. 2014; Steenekamp et al. 2021). However, no study has monitored both sleep‐wake patterns and sleep architecture in relation to changes in morning schedules and training load during a training camp. Such an in situ investigation may clarify how the sleep of rugby union players is affected during this typical training context.

The present study aimed to evaluate the sleep‐wake patterns and sleep architecture of young elite rugby union players during a 10‐day training camp, while considering the changes in daily physical demands and morning appointment times. We hypothesised that early morning appointment may impair sleep duration, and that the rugby‐specific physical demands have variable effects on sleep‐wake patterns and sleep architecture.

## 2 Materials and Methods

### 2.1 Participants

Twenty‐six elite male under‐20 rugby union players, that is, 14 forwards (mean ± SD; age: 18.8 ± 0.2 years; body mass = 104.7 ± 8.7 kg; height = 188.2 ± 7.5 cm) and 12 backs (age: 18.6 ± 0.4 years; body mass = 84.4 ± 7.8 kg; height = 178.7 ± 5.3 cm), from various clubs, participated in the protocol. Players were classified at ‘Elite/International Level’ (Tier 4) according to the guidelines proposed by (McKay et al. 2022), as they were all competing at the highest national level and members of the national team. Players were excluded (*n* = 0) in the case of medical circumstances influencing training frequency, taking medication for sleep disorders, and travel involving crossing of several time zones in the 2 weeks preceding the protocol. Before beginning, they received comprehensive verbal and written explanations of the purpose of the study and the experimental procedure, and all provided written informed consent. The study was approved by the local ethic committee (approval reference: 21.00586.000020, ID‐RCB: 2021‐A01607‐34) and was conducted in accordance with the Declaration of Helsinki (1964: revised in 2001).

### 2.2 Training Camp

**FIGURE 1:**
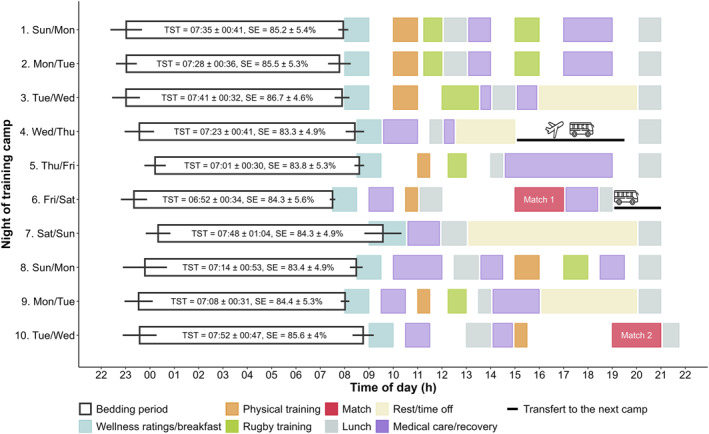
Sleep‐wake patterns according to the training camp timeline. Each line represents a 24‐h training camp day from 22:00 to 22:00. White bars indicate the mean ± SD start and end times of bedding periods. Coloured bars indicate the day's scheduled events. Data are reported as mean ± SD. SE, sleep efficiency; TST, total sleep time (hh:min).

Sleep‐wake patterns, sleep architecture, training load, and perceived sleep quality and wellness were monitored on a daily basis during a 10‐day training camp as part of a national team convocation, interspersed with two exhibition matches (days 6 and 10). The training camp took place in April 2023. For days 1–4, it was held at a location where all players were accustomed to training and sleeping. The camp then moved to two different locations for days 5–6 and days 7–10, without any change in time zone. The first journey was completed by plane and bus (4 h), and the second by bus only (2 h). The program was developed by the team coaches (Figure 1) and consisted of 6 training days (days 1–3, 4, 8–9), two rest days (4, 7) and 2 exhibition match days (days 6, 10), kicking off at 15:00 and 19:00, respectively. Morning appointment time varied from 7:30 (*n* = 1), 8:00 (*n* = 4), 8:30 (*n* = 3) to 9:00 (*n* = 2). The training program for days 1–3 focused on high‐intensity training sessions including collisions. Then, a reduction in intensity and number of training sessions was emphasised, interspersed with the two exhibition matches. At the same time, the number of rest and time off periods increased. Players had free choice of bedtime and slept in shared twin rooms with separate beds, the pairing remained unchanged throughout the camp. Each morning they had a 1‐h window, that is, morning appointment time, during which they rated their perceived sleep quality and wellness (i.e., general fatigue, pain and stress), and ate breakfast. Following the lunch break, players had a rest period (from 30 min to 1 h) and the possibility of taking a nap. Players were permitted to consume caffeine and refrained from alcohol or sleep medication during the camp.

At the start of the training camp, all players were asked to complete the following common sleep questionnaires: (a) the Morningness‐Eveningness Questionnaire (MEQ) to identify the individual's chronotype (Horne and Ostberg 1976), (b) the Epworth Sleepiness Scale (ESS) to assess daytime sleepiness (Johns 1991), (c) the Pittsburgh Sleep Quality Index (PSQI) to evaluate the perceived sleep quality (Buysse et al. 1989), (d) the Ford Insomnia Response to Stress Test (FIRST) to measure the vulnerability of sleep disturbance in response to common stressors (Drake et al. 2004) and (e) the Insomnia Severity Index (ISI) to assess severity of insomnia symptoms (Morin et al. 2011). While these questionnaires were originally developed for use with the general population (Ibáñez et al. 2018), they are often used in elite athletes as a first‐line diagnostic tool to identify poor sleep habits and potential sleep disorders (Halson 2019).

Players also received wristwatch actigraphy worn from before going to bed to getting up in the morning to record sleep‐wake patterns and an electronic sleep diary in which to report the daily perceived sleep quality and wellness. No data were monitored after the second match, which marked the end of the training camp and the return of the players to their respective clubs. To assess sleep architecture, 13 forward players also received an electroencephalographic (EEG) headband, with the first night considered as a familiarisation (Hof zum Berge et al. 2021) and not considered for further analysis. We chose to equip only the forward players because of a limited number of EEG headbands. Forward players are also more likely to experience collisions than back players (Naughton et al. 2020), which may subsequently influence sleep architecture (Aloulou et al. 2020).

### 2.3 Training Load Quantification

#### 2.3.1 External Training Load

Players wore a portable micro‐electromechanical system (mass: 67 g, size: 88 × 50 × 19 mm, MinimaxXTM S4, Catapult Innovations, Australia) during each rugby training and match session. This tool contains a 10‐Hz Global Positioning Systems (GPS), a 100‐Hz triaxial accelerometer, a gyroscope and a magnetometer which provide access to the body centre of mass position, movement velocity and acceleration as a function of time. The device was positioned in a Lycra vest on the upper thoracic spine between the scapulae. To avoid any inter‐device variability, players kept the same device throughout the training camp (Coutts and Duffield 2010). Total and relative distance covered per minute, at low‐ (< 18 km.h^−1^) and high‐speed (HSD, > 18 km.h^−1^), and high‐intensity acceleration (≥ 2.5 m.s^−2^) and deceleration (≤ −2.5 m.s^−2^) were used to assess the locomotor load according to previously‐established thresholds (Lacome et al. 2017; Harper et al. 2019). These external training load metrics represent the most important components associated with rugby union‐related metabolic and neuromuscular fatigue (Naughton et al. 2021). The accuracy and reliability of GPS units to measure these movement patterns have been demonstrated as ‘acceptable’ in team sports (Varley et al. 2012; Rampinini et al. 2015; Scott et al. 2016). Finally, collision events have been detected by the device through changes in axis orientation and impact forces > 8 *g*. These detection have been demonstrated to be accurate (93%), sensitive (94%), and specific (93%) in identifying collision events in professional rugby union players (MacLeod et al. 2018). On rest days, external training loads metrics were scored as 0.

#### 2.3.2 Perceived Training Load

The session rating of perceived exertion (sRPE) was recorded within 30 min from the end of each training session using the modified CR‐10 scale (Foster et al. 2001), which players were all accustomed to. The daily perceived training load (A.U.) was calculated by multiplying sRPE and the duration of each training session within a day (sRPE × training duration). This method has been shown to be reliable for assessing internal loads in rugby (Lovell et al. 2013). On rest days, internal training load was scored as 0.

### 2.4 Sleep Monitoring

#### 2.4.1 Actigraphy

Actigraphy data were collected using an activity monitor (MotionWatch 8; CamNtech LtD, Cambridge, UK) worn on the nondominant wrist. The recorded data were analysed using MotionWare Software (version 1.3.17; CamNtech LtD, Cambridge, UK) to identify whether the players were awake or asleep for each 60‐s period. Actigraphy is a low‐cost and non‐invasive method for longitudinal monitoring sleep‐wake patterns that has been shown to generate the smallest mean biases from polysomnography for sleep duration, sleep efficiency and WASO in team‐sport athletes when a medium sleep/wake sensitivity threshold (> 40 activity counts are scored as wake) is applied (Fuller et al. 2017). A medium sensitivity sleep/wake threshold was accordingly applied to the present population of team‐sport athletes. Actigraphic sleep variables were measured as follows: bedtime (h:mm), the self‐reported clock time when the player attempts to sleep; get‐up time (h:mm), the self‐reported clock time when he gets out of bed and stops attempting to sleep; time in bed (TIB − min), the time between bedtime and get‐up time; total sleep time (TST − min), the time spent asleep, determined from sleep onset to sleep offset, minus any waking time; WASO (min), the amount of time spent awake between sleep onset and sleep offset; sleep efficiency (%), the TST expressed as a percentage of TIB; sleep onset latency (SOL − min), the time between bedtime and sleep onset. When the self‐reported bedtime and/or get‐up time did not match that of the sleep diary, the night was excluded.

#### 2.4.2 EEG Headband

Sleep architecture was assessed using a reduced‐montage dry‐EEG headband device (Dreem 3, Dreem, Paris, France). Players were instructed to place the device on their head just before going to bed and remove it following get‐up time. The device is a wireless headband which records and stores four types of sleep‐related physiological signals in real time: (1) brain cortical activity via five EEG dry electrodes with high‐consistency silicone with soft, flexible protuberances yielding five derivations (F7, F8, Fp1, O1, O2 according to the International 10–20 system; 250 Hz with a 0.4–35 Hz bandpass filter), and (2–4) movements, head position and breathing frequency by a 3D accelerometer located over the headband. A proprietary algorithm enables automatic sleep stage analysis in two phases: (1) feature extraction in 30‐s epochs from the relative EEG spectral power computed in frequency bands relevant for sleep analysis (*λ* = 0.5–4 Hz, *θ* = 4–8 Hz, *α* = 8–14 Hz and *β* = 15–30 Hz), and (2) classification to establish a hypnogram from 30‐s epochs scored as wake or sleep stages (i.e., light sleep: N1 + N2, SWS or rapid eye movement—REM sleep). The signal acquisition of the EEG headband and the performance of its embedded automatic sleep staging algorithms have been validated by the manufacturer's scientific team compared to the consensus of five independent sleep specialists using a medical‐grade polysomnography (Arnal et al. 2020). Automatic sleep‐staging classification performed by the EEG headband was similar (overall accuracy: 83.5 ± 6.4%) to the inter‐scorer accuracy achieved with PSG by sleep specialists (overall accuracy: 86.4 ± 8.0%).

The EEG headband recordings were then processed as follows to select the best‐quality recordings, using a similar approach from (Knufinke et al. 2018): (1) bedtime and get‐up time were adjusted according to actigraphy and sleep diaries as reference; (2) if there were missing epochs at bedtime and get‐up time, they were manually added as ‘undefined’; (3) all ‘undefined’ epochs prior to sleep onset were manually scored as ‘wake’; (4) REM sleep epochs within the first 30 min of the recording without succeeding SWS were manually scored as ‘undefined’ to be consistent with the normative range of REM sleep latency (49.5–278.5 min; (Mitterling et al. 2015)); and (5) recordings containing more than 15% ‘undefined’ epochs were excluded. Sleep architecture was determined by calculating the proportion of time spent in each sleep stage (i.e., light sleep, SWS and REM sleep—%) relative to the total time spent in all sleep stages. The number of sleep stage shifts was calculated as the number of transitions from one sleep stage to another or to wakefulness, expressed per hour of sleep.

### 2.5 Perceived Sleep Quality and Wellness

Players were asked to rate their perceived sleep quality and wellness in a sleep diary via their smartphone during a 1‐hour window upon waking, using an online software for conducting surveys (Smartabase, 2023–2024, Teamworks Innovation, NC, Durham, USA). They reported their bedtime and get‐up time, and assessed their perceived sleep quality using the Spiegel Sleep Inventory (SSI). The SSI is composed of six questions scored from 1 to 5 about sleep initiation, the quality and duration of sleep, nocturnal awakenings, dreams, and feeling refreshed in the morning. The overall score is the sum of the six items, and a higher score indicates a better sleep quality. Few data are available on the psychometric validity of the SSI among athletes, but it is a very simple and easy‐to‐use scale that is often used to assess the presence of insomnia (Léger et al. 2006). Perceived general fatigue, pain (including muscle soreness) and stress were also rated during a 1‐hour window upon waking in the sleep diary using a visual analogue scale from 0 to 10, with a sliding cursor (from ‘no pain’, ‘no fatigue’, or ‘no stress’ to ‘unbearable pain’, ‘very, very tired’, or ‘very, very stressed’).

### 2.6 Statistical Analysis

Data processing and statistical analyses were conducted using R software (version 4.3.2). The proportion of nights outside the National Sleep Foundation's recommendations (Hirshkowitz et al. 2015; Ohayon et al. 2017) were reported as percentage. The between‐playing positions comparisons in sleep questionnaire scores were conducted using a Student *t*‐test comparison. The chi‐squared test was used to compare chronotype proportions between the playing positions. No obvious deviations from normality of data distribution and the homogeneity of variance were reported using the Shapiro–Wilk and Levene tests, respectively.

Sleep data (i.e., actigraphic sleep variables, sleep architecture and SSI score) and wellness ratings (i.e., general fatigue, pain and stress) over the training camp were analysed using linear mixed‐effects models (‘lmer’ function of the ‘lme4’ R package) to take into account the potential missing data and the non‐independence of observations at the players level (Harrison et al. 2018). All models were estimated including the player as a random effect. The between‐playing position comparisons in actigraphic sleep variables and SSI score were conducted using a first model including the playing position as fixed effect. A second linear mixed‐effects model was performed to assess the relationship between sleep‐wake patterns, SSI score and the following variables as fixed effects: the morning appointment time (categorical predictor) and the prior training load metrics as continuous predictors (i.e., HSD, number of collisions, number of high‐intensity accelerations and high‐intensity decelerations, and sRPE × training duration). A separate linear mixed‐effects model was used to evaluate the relationship between sleep architecture among forward players and the same prior training load covariates, adjusted for the morning appointment time. The significance of the morning appointment time effect was determined using the ‘mixed’ function from the ‘afex’ R package, using type 2 test. Post‐hoc Student *t*‐test pairwise comparisons were conducted when a significant main effect was reported, using least square means contrasts, with the ‘Tukey’ method for alpha adjustments and a Kenward‐Roger adjustment for the degrees of freedom calculation (‘emmeans’ function of the ‘emmeans’ package). The effect sizes (ES) and their 90% confidence intervals (CI) were calculated to interpret the magnitude of the difference in means values, and were interpreted using the following criteria: < 0.2 = *trivial*, 0.2–0.6 = *small*, 0.6–1.2 = *moderate*, 1.2–2.0 = *large* and > 2.0 = *very large* (HOPKINS et al. 2009). A third linear mixed‐effects model was performed to evaluate the relationships between sleep‐wake patterns, sleep architecture, SSI score, and perceived general fatigue, pain and stress upon waking (fixed effects). Variance inflation factors were used to assess collinearity issues between variables in all models, and all were < 6, indicating a low collinearity. For each linear mixed‐effects model, the normality of residuals was visually checked using a Q–Q plot, and no obvious deviations from normality were revealed.

The descriptive data are reported as the mean ± SD, and results from the linear mixed‐effects models are presented as the estimate (Est.), 95% confidence interval (CI) and *p*‐value. The level of significance was set at *p* < 0.05.

## 3 Results

### 3.1 Sleep Questionnaires

The mean ± SD scores from sleep questionnaires were below the cut‐off suggesting sleep complaints (ESS: 9.5 ± 4.4 A.U.; PSQI: 3.5 ± 1.4 A.U.; FIRST: 16.5 ± 4.8 A.U.; ISI: 4.9 ± 3.9 A.U.), with no significant differences between playing position (*p* > 0.51). One player reported perceived ‘poor sleep quality’ and one other player reported ‘moderate insomnia’ based on global PSQI score ≥ 5 and ISI score ≥ 15, respectively. Ten players indicated that they experienced excessive daytime sleepiness (ESS score ≥ 10). The MEQ revealed that players were either definitely morning type (*n* = 1), moderately morning type (*n* = 6), neither type (*n* = 15) or moderately evening type (*n* = 2) with no significant differences between playing positions (*p* = 0.25).

### 3.2 Sleep During the Training Camp

The 10‐day training camp resulted in a total of 198 out of 260 (i.e., 76.2%) possible actigraphy‐based sleep recordings, with missing data due to participants forgetting to wear the actigraphy of some nights or conflicts between recorded bedtimes and wake‐up times and those in the sleep diary. A total of 45 out of 117 (i.e., 38.5%) possible EEG headband‐based sleep recordings was obtained. Missing data from the EEG headband were due to mismatches with bedtimes and get‐up times from actigraphy and sleep diaries, recordings containing more than 15% ‘undefined’ epochs, or players not wearing the EEG headband. One player did not present actigraphy data and two other players did not show EEG headband data throughout the camp.

Average sleep data over the training camp according to playing positions are presented in Table 1. Overall, 30.3% of nights showed a TST < 7 h, 77.8% had WASO > 40 min, 43.4% had sleep efficiency < 85% and 20.7% had SOL > 30 min. The average TST was > 7 h for all nights (mean ± SD: 07:24 ± 00:44 h), except for the night prior to Match 1, during which the players obtained 06:52 ± 00:34 h of sleep, alongside the earliest get‐up time (7:30 ± 00:06, Figure 1). No significant differences between forward and back players were observed in any sleep‐wake patterns, except for TIB, which was longer for the back players than for the forward players (+18.6 min, ES = 0.49, *p* = 0.03).

**TABLE 1:** Descriptive statistics (mean ± SD) of sleep data over the training camp according to playing positions.

	Overall	Backs	Forwards	Playing position differences
Sleep‐wake patterns				
Bedtime (h:min)	23:29 ± 00:44	23:24 ± 00:43	23:32 ± 00:44	ES = 0.23 (*small*), *p* = 0.37
Get‐up time (h:min)	08:13 ± 00:36	08:18 ± 00:33	08:09 ± 00:37	ES = −0.27 (*small*), *p* = 0.16
TIB (h:min)	08:44 ± 00:41	08:54 ± 00:38	08:37 ± 00:42	ES = −0.49 (*small*), ***p* = 0.03**
TST (h:min)	07:24 ± 00:44	07:27 ± 00:44	07:21 ± 00:43	ES = −0.24 (*small*), *p* = 0.31
WASO (min)	56.7 ± 19.7	60.2 ± 22	54.2 ± 17.5	ES = −0.29 (*small*), *p* = 0.59
Sleep efficiency (%)	84.7 ± 5.1	83.8 ± 5.7	85.4 ± 4.5	ES = 0.26 (*small*), *p* = 0.46
SOL (min)	20.3 ± 20.6	21.8 ± 22.6	19.2 ± 18.9	ES = −0.13 (*trivial*), *p* = 0.51
SSI Score (A.U.)	22.8 ± 3.1	23.3 ± 3.3	22.3 ± 2.9	ES = −0.41 (*small*), *p* = 0.13
Sleep architecture				
Light sleep (N1 + N2—%)	—	—	51.0 ± 6.6	—
SWS (%)	—	—	24.1 ± 6.8	—
REM (%)	—	—	25.0 ± 5.0	—

*Note* *:* Bold indicates statistically significant at *p* < 0.05.

Abbreviations: A.U., arbitrary unit; ES, effect size; N1–N2, NREM sleep stages 1–2; REM, rapid eye movement sleep stage; SOL, sleep onset latency; SSI, Spiegel sleep inventory; SWS, slow wave sleep; TIB, time in bed; TST, total sleep time; WASO, wake after sleep onset.

### 3.3 Relationship Between Sleep, Daily Training Load and Perceived Wellness

Daily changes in training load metrics are shown in Table 2. Tables 3 and 4 provides an overview of the relationship between sleep and training load preceding the night and perceived wellness covariates, adjusted for the morning appointment time. Every additional 100‐m in HSD was associated with subsequent increases in TIB (*β* = +4.5 min [95% CI, 0.5 to 8.5 min], *p* < 0.05) and TST (*β* = +4.9 min [0.6 to 9.2 min], *p* < 0.05), as well as a reduction in the number of sleep stage shift per hour of sleep (*β* = −1.1 [−2.1 to −0.2], *p* < 0.05). Each additional 1 A.U. in general fatigue was associated with a later prior bedtime (*β* = +4.3 min [0.3 to 8.3 min], *p* < 0.05), an increase in WASO (*β* = +1.7 min [0.2 to 3.1 min], *p* < 0.05), and a reduction in SSI score (*β* = −0.6 A.U. [−0.8 to −0.4 A.U.], *p* < 0.001). No significant relationships were observed between the other sleep variables and training load, and perceived wellness covariates.

**TABLE 2:** Changes in training load metrics throughout the training camp.

Day	Total distance (m)	Relative distance (m.min^−1^)	HSD (m)	Collisions (*n*)	HIA (*n*)	HID (*n*)	sRPE × duration (A.U)
1. Monday	2934.4 ± 1189.5	39.8 ± 16.1	170.9 ± 137.1	11.5 ± 7.3	17.7 ± 9.9	13.3 ± 6.8	449.3 ± 174.9
2. Tuesday	3620.2 ± 417.9	57.6 ± 6.6	441.0 ± 174.7	12.5 ± 5.8	17.9 ± 8.8	10.3 ± 6.0	551.4 ± 62.7
3. Wednesday	3710.5 ± 487.9	49.6 ± 7.8	370.8 ± 209.9	15.4 ± 6.0	19.3 ± 9.3	13.9 ± 7.1	702.5 ± 93.9
4. Thursday	Rest						
5. Friday	2569.7 ± 315.3	56.5 ± 6.9	264.3 ± 70.8	7.7 ± 3.4	11.0 ± 6.0	7.3 ± 3.9	341.5 ± 64.6
6. Saturday (match)	5738.8 ± 1154.4	65.7 ± 6.7	599.5 ± 272.7	22.4 ± 8.2	28.2 ± 13.0	20.4 ± 10.9	660.1 ± 135.7
7. Sunday	Rest						
8. Monday	3382.4 ± 467.7	42.3 ± 5.9	161.5 ± 101.2	5.8 ± 5.0	18.0 ± 12.5	12.0 ± 8.5	545.4 ± 95.7
9. Tuesday	2673.5 ± 202.1	59.4 ± 4.3	253.5 ± 75.8	7.8 ± 4.1	14.8 ± 6.5	8.3 ± 4.1	355.4 ± 82.1
10. Wednesday (match)	5601.1 ± 1495.2	65.8 ± 7.5	516.7 ± 265.0	23.3 ± 12.3	31.5 ± 14.2	23.0 ± 11.5	674.4 ± 128.5

*Note*: Data are reported as mean ± SD.

Abbreviations: A.U., arbitrary unit; HIA, high‐intensity acceleration ≥ 2.5 m.s^−2^; HID, high‐intensity deceleration ≤ −2.5 m.s^−2^; HSD, high‐speed distance > 18 km.h^−1^; sRPE × duration, session‐rating of perceived exertion × training session duration.

**TABLE 3:** Relationship between sleep‐wake patterns and perceived sleep quality (SSI score), training load preceding the night and perceived wellness, adjusted for morning appointment times.

	Bedtime (min)		Get‐up time (min)		Time in bed (min)		Total sleep time (min)	
Fixed effects	Est. (95% CI)	*p*	Est. (95% CI)	*p*	Est. (95% CI)	*p*	Est. (95% CI)	*p*
Training load								
HSD (× 100‐m)	2.5 (−6.3 to 1.4)	0.21	1.9 (−0.4 to 4.2)	0.10	4.5 (0.5 to 8.5)	**0.03**	4.9 (0.6 to 9.2)	**0.03**
Collisions (*n*)	−0.8 (−1.9 to 0.3)	0.16	−0.1 (−0.7 to 0.6)	0.80	0.6 (−0.6 to 1.7)	0.34	0.3 (−0.9 to 1.5)	0.59
HIA and HID (*n*)	0.3 (−0.2 to 0.8)	0.28	−0.0 (−0.3 to 0.3)	0.87	−0.2 (−0.7 to 0.3)	0.39	−0.3 (−0.9 to 0.2)	0.21
sRPE × duration (× 100 A.U.)	0.5 (−3.2 to 4.3)	0.78	−0.7 (−2.9 to 1.5)	0.53	−1.4 (−5.4 to 2.5)	0.48	−0.8 (−5.0 to 3.4)	0.71
Morning appointment times (main effect)		**<** **0.001**		**<** **0.001**		**<** **0.001**		**<** **0.001**
Subjective ratings								
General fatigue (A.U.)	4.3 (0.3 to 8.3)	**0.04**	2.6 (−0.7 to 5.9)	0.12	−1.5 (−5.4 to 2.4)	0.45	−3.0 (−7.0 to 1.0)	0.14
Pain (A.U.)	1.0 (−4.4 to 6.3)	0.73	3.8 (−0.8 to 8.3)	0.11	3.0 (−2.2 to 8.2)	0.26	2.7 (−2.8 to 8.2)	0.34
Stress (A.U.)	0.2 (−6.9 to 7.2)	0.96	−2.6 (−8.4 to 3.2)	0.37	−2.9 (−9.8 to 4.1)	0.42	1.0 (−6.1 to 8.1)	0.79

	Wake after sleep onset (min)		Sleep efficiency (%)		Sleep latency (min)		SSI score (A.U.)	
Fixed effects	Est. (95% CI)	*p*	Est. (95% CI)	*p*	Est. (95% CI)	*p*	Est. (95% CI)	*p*
Training load								
HSD (× 100‐m)	0.8 (−0.8 to 2.4)	0.31	0.3 (−0.2 to 0.8)	0.21	−0.8 (−2.9 to 1.4)	0.49	−0.1 (−0.3 to 0.2)	0.73
Collisions (*n*)	0.2 (−0.2 to 0.7)	0.29	−0.0 (−0.2 to 0.1)	0.69	−0.1 (−0.7 to 0.6)	0.85	−0.0 (−0.1 to 0.1)	0.96
HIA and HID (*n*)	0.0 (−0.2 to 0.2)	0.69	−0.0 (−0.1 to 0.0)	0.41	−0.1 (−0.3 to 0.2)	0.63	0.0 (−0.0 to 0.1)	0.25
sRPE × duration (× 100 A.U.)	−0.6 (−2.1 to 0.8)	0.39	0.0 (−0.5 to 0.5)	0.97	0.5 (−1.6 to 2.7)	0.63	−0.1 (−0.4 to 0.1)	0.35
Morning appointment times (main effect)		0.25		**0.01**		0.43		0.62
Subjective ratings								
General fatigue (A.U.)	1.7 (0.2 to 3.1)	**0.02**	−0.2 (−0.7 to 0.2)	0.34	−0.2 (−2.0 to 1.7)	0.86	−0.6 (−0.8 to −0.4)	**<** **0.001**
Pain (A.U.)	0.1 (−1.6 to 1.8)	0.92	−0.0 (−0.6 to 0.5)	0.88	0.1 (−2.5 to 2.7)	0.93	0.2 (−0.2 to 0.5)	0.31
Stress (A.U.)	−2.5 (−5.0 to −0.0)	**0.05**	0.6 (−0.2 to 1.4)	0.15	−1.7 (−4.9 to 1.5)	0.29	0.1 (−0.2 to 0.5)	0.49

*Note* *:* Bold indicates statistically significant at *p* < 0.05.

Abbreviations: A.U., arbitrary unit; CI, confidence interval; Est., estimate; HIA, high‐intensity acceleration ≥ 2.5 m.s^−2^; HID, high‐intensity deceleration ≤ −2.5 m.s^−2^; HSD, high‐speed distance > 18 km.h^−1^; sRPE × duration, session‐rating of perceived exertion × training session duration; SSI, Spiegel sleep inventory.

**TABLE 4:** Relationship between sleep architecture, training load preceding the night and perceived wellness, adjusted for the morning appointment time.

	Light sleep (%)		N2 (%)		SWS (%)		REM (%)		Sleep stage shifts (/h)	
Fixed effects	Est. (95% CI)	*p*	Est. (95% CI)	*p*	Est. (95% CI)	*p*	Est. (95% CI)	*p*	Est. (95% CI)	*p*
Training load										
HSD (× 100‐m)	−1.3 (−3.52−0.9)	0.25	−0.8 (−2.7 to 1.2)	0.42	0.3 (−1.7 to 2.2)	0.78	0.9 (−0.7 to 2.6)	0.25	−1.1 (−2.1−0.2)	**0.03**
Collisions (*n*)	0.0 (−0.4 to 0.5)	0.85	−0.0 (−0.4 to 0.4)	0.96	−0.0 (−0.4 to 0.4)	0.92	−0.0 (−0.3 to 0.3)	0.93	0.2 (−0.0 to 0.4)	0.08
HIA and HID (*n*)	0.0 (−0.3 to 0.3)	0.91	0.0 (−0.2 to 0.3)	0.77	−0.1 (−0.3 to 0.1)	0.40	0.0 (−0.2 to 0.2)	0.76	−0.0 (−0.2 to 0.1)	0.52
sRPE × duration (× 100 A.U.)	0.6 (−1.1 to 2.2)	0.48	0.3 (−1.1 to 1.7)	0.62	0.2 (−1.3 to 1.6)	0.82	−0.5 (−1.7 to 0.7)	0.40	0.3 (−0.5 to 1.0)	0.46
Morning appointment times (main effect)		0.70		0.58		0.69		0.17		0.52
Subjective ratings										
General fatigue (A.U.)	0.4 (−1.3 to 2.1)	0.60	0.4 (−1.1 to 1.8)	0.61	−0.9 (−2.4 to 0.5)	0.21	0.4 (−0.9 to 1.7)	0.50	0.3 (−0.5 to 1.1)	0.51
Pain (A.U.)	0.5 (−1.6 to 2.7)	0.62	0.5 (−1.4 to 2.3)	0.61	0.3 (−1.7 to 2.2)	0.79	−0.7 (−2.4 to 1.0)	0.40	0.2 (−0.9 to 1.3)	0.69
Stress (A.U.)	−0.9 (−3.0 to 1.1)	0.35	−0.7 (−2.4 to 1.0)	0.43	0.6 (−1.2 to 2.5)	0.49	0.2 (−1.3 to 1.7)	0.79	−0.4 (−1.4 to 0.6)	0.42

*Note* *:* Bold indicates statistically significant at *p* < 0.05.

Abbreviations: A.U., arbitrary unit; CI, confidence interval; Est., estimate; HIA, high‐intensity acceleration ≥ 2.5 m.s^−2^; HID, high‐intensity deceleration ≤ −2.5 m.s^−2^; HSD, high‐speed distance > 18 km.h^−1^; sRPE × duration, session‐rating of perceived exertion × training session duration; SWS, slow‐wave sleep; REM, rapid eye movement.

### 3.4 Changes in Sleep‐Wake Patterns Depending on Morning Appointment Times

**FIGURE 2:**
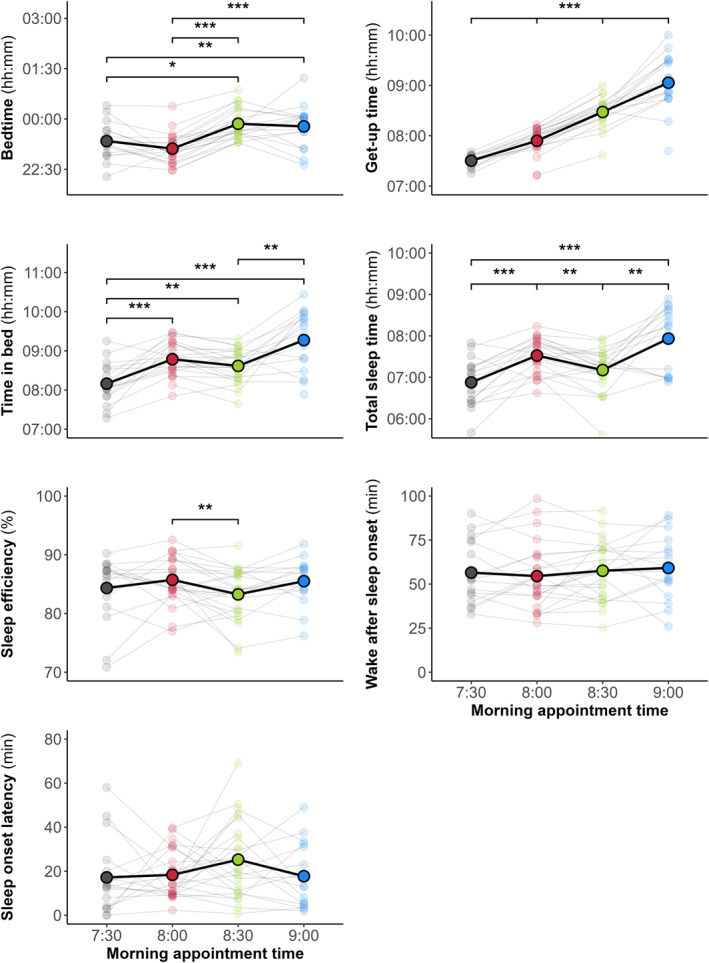
Changes in sleep‐wake patterns based on morning appointment times. Data are averaged over players, with individual data displayed. Significant post‐hoc differences are shown, *p < 0.05, **p < 0.01, ***p < 0.001.

The sleep‐wake patterns were compared across morning appointment times (Figure 2). A main effect of the morning appointment time was found for bedtime (F_(3, 171)_ = 29, *p* < 0.001), get‐up time (F_(3, 169)_ = 137, *p* < 0.001), time in bed (F_(3, 171)_ = 12, *p* < 0.001), total sleep time (F_(3, 174)_ = 10, *p* < 0.001) and sleep efficiency (F_(3, 171)_ = 4, *p* = 0.01). Delaying the morning appointment time from 7:30 to 9:00 resulted in progressively later bedtimes and get‐up times, and an increase in TIB (*p* < 0.05). In contrast, a *small* impairment in TST (−19.3 min [95% CI, −35.8 to −2.8 min], ES = −0.53, *p* = 0.01) and sleep efficiency (−2.2% [−4.0% to −0.5%], ES = −0.57, *p* < 0.01) was observed when the subsequent morning appointment time was scheduled at 8:30 compared to 8:00. The lowest TIB and TST occurred the night prior to the earliest appointment time scheduled at 7:30 (*p* < 0.05). No main effect of the morning appointment time was found for the other sleep‐wake patterns and sleep architecture variables (Tables 3 and 4).

## 4 Discussion

The present study revealed that (1) players encountered difficulties in achieving at least 7 h of sleep per night and experienced elevated WASO during the training camp compared to the National Sleep Foundation's recommendations for healthy young adults, (2) higher distance covered at high speed promotes subsequent sleep duration and continuity of sleep architecture and (3) the morning appointment time determines the sleep‐wake patterns of the previous night.

The results of the present study showed a substantial prevalence of nights characterised by a TST < 7 h (30.3%), WASO > 40 min (77.8%) and sleep efficiency < 85% (43.4%), that is, outside the National Sleep Foundation's recommendations for healthy young adults (Hirshkowitz et al. 2015; Ohayon et al. 2017). Previous studies also observed an impairment in sleep‐wake patterns, that is, TST, WASO and/or sleep efficiency, among players undergoing a training camp in team‐based contact sports (Pitchford et al. 2017; Thornton et al. 2017). Factors such as unfamiliar sleeping environment, training load (Pitchford et al. 2017), sharing a bedroom with a teammate (Costa et al. 2023), and unaccustomed and congested schedules (e.g., several training sessions a day, meetings, working sessions and collective lunches, travels), all observed in the present study, have been identified as potential reasons why camp can be a vulnerable period for athletes' sleep (Nedelec et al. 2018). However, the players’ sleep was not monitored in their usual sleep environment before the camp, so we cannot conclude whether the training camp altered their sleep patterns or if the players are simply poor sleepers.

According to the literature, we hypothesised that the rugby‐specific physical demands have variable effects on sleep‐wake patterns and sleep architecture. The present findings indicate that, irrespective of the morning appointment time effect, every additional 100‐m of HSD increases the subsequent TIB (+4.5 min) and TST (+4.9 min). A novelty of this study is the introduction of the EEG headband, which revealed a reduction in the number of sleep stage shifts following higher daily HSD. These findings support the evidence that higher running demands promote sleep propensity and the continuity of sleep architecture (Aloulou et al. 2020; Chauvineau et al. 2023). Conversely, we did not demonstrate detrimental effects of rugby‐specific physical demands on subsequent sleep‐wake patterns and sleep architecture, as reported elsewhere (Thornton et al. 2017, 2018; Leduc et al. 2019; Aloulou et al. 2020). Possible explanations for this discrepancy may be related to the training load achieved and/or the training period. For instance, Thornton et al. (2017) observed a *moderate* negative within‐player correlation between prior running total distance and TST (*r* = −0.3 ± 0.2), when rugby league players were exposed to a 2‐week preseason high‐intensity training camp. The quantity and subjective quality of sleep of international rugby seven players have been also shown to be impaired during weeks with highest training loads, especially with regards to the total distance covered, HSD, number of accelerations and decelerations, and perceived training load (Leduc et al. 2019). Moreover, Aloulou et al. (2020) observed a negative impact of the number of collisions (assessed via video analysis) experienced by players during an official rugby‐union match on subsequent sleep architecture. In the present study, the daily rugby‐specific physical demands were clearly below those in previous studies, mainly because the camp program was designed to prepare players for two exhibition matches in a congested period of time (i.e., 5 days) without accumulating excessive fatigue. An inverted U‐shaped, dose–response relationship model between training load and quantity and/or quality has been proposed (Falck et al. 2021). This model suggests that increases in training load promote sleep until a ‘tipping point’, beyond which additional increases may result in sleep disturbances. It is therefore possible that the training load was well managed, not exceeding a threshold that would lead to sleep impairment. As such, coaches could take this result into account to maintain a controlled and not excessive training load, for preserving sleep quantity and quality. Future studies are required to further investigate how various physical demands may affect sleep (especially its architecture), when rugby‐union players are exposed to high training loads, for example, during an intensified preseason or tournament preparation period.

Another major finding from this study was that sleep‐wake patterns changed according to the morning appointment time. Some studies have demonstrated that morning schedule strongly determines the prior sleep duration (Sargent, Lastella, et al. 2014; Sargent, Halson and Roach 2014; Steenekamp et al. 2021). From various sports, Sargent, Lastella, et al. (2014) observed that the earlier the training start time from 8:00 to 5:00, the less sleep athletes obtained the night before. Although the morning training sessions were scheduled for a later time in the present study (starting from 10:00), players had appointments scheduled between 7:30 and 9:00, depending on the day, to start the day collectively. The earliest scheduled morning appointment, that is, 7:30, was associated with the shortest average sleep duration (06:52 ± 00:34 h), which falls below the recommendations for healthy young adults (Ohayon et al. 2017). This sleep reduction may be at least partly explained by the fact that players did not compensate for having to wake up earlier by going to bed earlier. On the one hand, most social engagements are scheduled in the evening, which could limit the extent to which they can advance their bedtime. On the other hand, it is possible that sleep propensity was not enough for players to fall asleep earlier. Sleep propensity is driven by the interaction between a circadian process, which determines the sleep‐wake cycle, and a homeostatic process, which reflects the pressure for sleep that increases during wakefulness (Borbély et al. 2016). Sleep onset is most favourable during the circadian sleep phase and when sleep propensity is highest. Consequently, going to bed earlier in prevention of an earlier wake‐up may not ensure the preservation of sleep duration. This earlier morning appointment occurred the night before Match 1. Considering the importance of sleep for athlete preparation (Halson and Juliff 2017; O'Donnell et al. 2018) and the negative impact of sleep restriction on athletic performance (Craven et al. 2022), advancing the morning appointment time just before a match may not be conducive to achieving peak performance during a match. This issue is particularly important as poor sleep is common prior to competitions in team‐based contact sports, often due to nervousness and pre‐competition thoughts (Sargent et al. 2022; Sim et al. 2024). Furthermore, one unexpected result of the present study was that delaying the morning appointment time led players to getting up later, but it did not necessarily guarantee an increase in sleep duration or sleep efficiency. The highest average sleep duration (07:51 ± 00:54 h) was obtained when appointment time was scheduled at 9:00. Nevertheless, sleep duration (+19.3 min) and sleep efficiency (+2.2%) were the most favourable when morning appointments were scheduled at 8:00 compared to 8:30. This difference may be partly attributable to a greater delay in bedtime (+43.9 min) than in get‐up time (+33.5 min) at the 8:30 appointment compared to the 8:00 appointment. Players may have taken advantage of a later morning appointment to enjoy a longer free evening, which had a detrimental effect on sleep duration and efficiency.

Finally, our results indicate that delaying bedtime, experiencing prolonged WASO and impaired perceived sleep quality resulted in increased subsequent perceived fatigue. On the one hand, there is evidence that abrupt changes in sleep timing lead to disruption of the endogenous circadian system which may favour increased daytime fatigue (Boivin et al. 2022). On the other hand, delaying bedtime and prolonged WASO during the night could contribute to a reduced amount of sleep as well as an impairment in perceived sleep quality. Many athletes consider sleep as a flexible commodity that can be sacrificed for activities they perceive as more important or more enjoyable (Halson 2016). Bedtime procrastination, defined as failing to go to bed at the intended time despite the absence of external constraints, has been shown to contribute to perceived insufficient sleep quality and quantity, as well as increased daytime fatigue (Kroese et al. 2014; Kadzikowska‐Wrzosek 2018). These results support the importance of regular sleep habits, which has been demonstrated as an important factor for greater sleep duration and sleep efficiency in elite team sport athletes (Halson et al. 2022).

### 4.1 Limitations

Some limitations need to be considered when interpreting the present findings. The descriptive nature of the current study challenges the application of the results to other disciplines and/or other phases of the season (e.g. in‐season). However, exploring this type of period in elite athletes is very challenging. Importantly, the use of such a sleep methodology makes the results unique and in situ evidence remains of major interest in the context of high performance.

In addition, we did not monitor sleep during a usual training home period. We cannot exclude the potential effect of an unfamiliar sleep environment on the present results, as the training camp took place in three different locations. We also did not assess how unusual the training load may have been, nor the variability in training times and the daily program (e.g. meal times, rest/medical periods), all of which are important factors likely to affect sleep (Pitchford et al. 2017; Roberts et al. 2019). Nevertheless, the present investigation allows us to appraise the global impact of such contextual factors on sleep in elite athletes. It is also important to note that this study was carried out during a national team convocation, with putative high stakes for subsequent selection for major competitions. Of note, both exhibition matches were won during the present experiment which shows that the study did not have any detrimental effect on resulting performance. However, matches may have led to additional nervousness that could affect sleep (Juliff et al. 2015).

Furthermore, for practical reasons, players' well‐being was assessed solely through subjective measures using visual analogue scales. Players also did not provide any data on naps despite some of them napped, which would have been relevant to consider given the potential effect of napping on subsequent sleep during a camp (Thornton et al. 2017). Finally, some data were missing, particularly from the EEG headband. Linear mixed‐effects models were used to mitigate this methodological drawback and to assess the relationship between training load and sleep at the individual level while dealing with missing data (Harrison et al. 2018).

## 5 Practical Applications

When designing a training camp, our results encourage coaches to maintain a controlled and not excessive training load to preserve both the sleep quantity and quality of players. Coaches should also be aware of the putative negative impact of earlier and/or unusual morning appointment time on sleep, especially the day before or the day of a match. In particular, consistent morning appointment time should promote players' sleep quantity and quality and prevent perceived fatigue. It could be useful to implement sleep‐hygiene training courses for players, to promote consistent sleep timing and sleep quantity and quality during this compromised period for sleep.

## 6 Conclusion

The present study highlights the difficulty some rugby union players have in achieving adequate sleep quantity and their experience of elevated WASO during a training camp. In this context, daily moderately intensified training load had no detrimental effects on sleep. Conversely, higher running demands promoted sleep duration and the continuity of sleep architecture. Sleep‐wake patterns are strongly sensitive to changes in the timing of the morning appointment. Earlier and/or unusual morning appointment times reduced sleep duration and sleep efficiency.

## Ethics Statement

The study was approved by the local ethic committee (approval reference: 21.00586.000020, ID‐RCB: 2021‐A01607‐34) and was conducted in accordance with the Declaration of Helsinki (1964: revised in 2001).

## Consent

Informed consent was obtained from all participants included in the study.

## Conflicts of Interest

The authors declare no conflicts of interest.

## Data Availability

The data that support the findings of this study are available from the corresponding author upon reasonable request.
